# Developments in the philosophy of automatic analysis

**DOI:** 10.1155/S146392467800005X

**Published:** 1978

**Authors:** Peter B. Stockwell

**Affiliations:** Laboratory ofthe Government Chemist Cornwall House Stamford Street London SE1 9NQ UK


### Developments in the philosophy of automatic analysis*

**Peter B Stockwell**

*Laboratory of the Government Chemist, Cornwall House, Stamford Street, London, SE1 9NQ, UK.*

SINCE the 1950's automatic analysis has found increasing applications in industrial process control where both offline and online analysers have had considerable success. The rapid developments made in electronics, and subsequently computers, has enabled automation to be extended to the laboratory situation. Automatic methods of analysis, be they on commercial instruments or apparatus constructed internally, have thus become firmly established as common-place features of many analytical laboratories. This is particularly true of the clinical laboratory where, since the introduction of continuous flow analysis by Skeggs in 1957 [1], automatic analysers have eased the considerable bottle necks caused by the increased requirements of health care. However, for industrial applications automation is very much in an embryonic state. This is attributable to the complexity of the sample matrix. In clinical chemistry the samples are predictable being blood and urine for the most part which are fairly similar in composition. In process control situations the compositional variation between samples is normally slight and for the most part predictable. There is therefore an established market, both actual and potential, for automation in these two areas. For many industrial applications the sample matrix differs widely both within a single industry and even more so from one to another. Matrix problems often negate the simple transfer of say a clinical methodology into the industrial environment. As such, the majority of applications of automation in this sector of the industry relate specifically to the application of computer data processing, more recently with the emphasis on microprocessor based electronics, or to the simple mechanisation of a single aspect of the automatic regime; for example, an automatic syringe sampler for injection on to a gas chromatographic column.

Technological progress, in terms of equipment design and increased range of applicability, has been impressive. The techniques of discrete sample automatic analysis and of air-segmented continuous flow (the Technicon approach [1]) which have formed the basis of the vast majority of automated analytical systems have been augmented in recent years by centrifugal analysers (GeMSAEC [2]) and flow injection analysers [3]. Whilst at first sight the situation seems healthy it is fairly clear that the recent rapid advances have served to highlight significant problems which were only partially appreciated if at all in the early stages.

Automated equipment like any instruments must be justified in economic terms. Basically by increased efficiency of the laboratory operation or increased capacity and throughput of samples but more specifically by cost effectiveness. The principal concern of senior management is to ensure that these criteria are met. However, rather little constructive thought has been given to an equally important consequence, that automation, particularly on the large scale, brings about a fundamental change in the role of the analytical chemist. In manual analysis, the analytical chemist has control of the entire procedure and this personal association does much to generate a pride in the quality of the work as well as confidence in the results. When the same analytical chemist is operating an automatic analyser his role when the equipment is functioning properly, is apparently limited to loading the samples and reading the results from a printer or similar data processor. Deans [4] points out that the analyst has often failed to accept automation because he feels that his job has been downgraded. It is now clearly recognised that the analytical chemist's role remains a vital one; as the need for his manipulative skills declines when automation is introduced, so his detailed chemical knowledge and judgement assume greater importance. He is best fitted to the task of defining the precise specification of an automatic instrument intended to replace a manual procedure of which he is familiar. In a similar vein management must re-examine its approach to the training of staff and the organisation of their laboratory to work in more automated surroundings.

The success and vitality of an analytical laboratory extensively committed to automated procedures depends on resolving problems of the above nature as well as those more directly connected with technique, instrumentation and quality of results. Quality and relevance of results are critical criteria for a high-throughput automated analytical laboratory and they can only be guaranteed by a close and continuing rapport between the analytical chemist who fully understands the procedures being used to produce the results and the customer who must be able to interpret them in the light of his requirements.

Little effort has indeed been made in these areas but perhaps more surprising is the limited effort made to solve the fundamental problems involved in automating an analysis. Here the real problem of handling samples has been avoided and the main thrust been made on measurement and data processing. This is accentuated because of a complete lack of definition of automatic analysis.

### What is automatic analysis?

'Automatic Analysis' is well established as a subject area but less well defined in terms of what that area embraces. The International Union of Pure and Applied Chemistry (IUPAC) has sought, through its Commission on Analytical Nomenclature, to offer rigorous definitions and to provide a common international terminology. Thus, *automation* is defined as 'the use of combinations of mechanical and instrumental devices to replace, refine, extend or supplement human effort and facilities in the performance of a given process, in which at least one major operation is controlled without human intervention, by a feed-back mechanism' and *mechanization* is 'the use of mechanical devices to replace, refine, extend or supplement human effort'. The distinction between the two terms is clear. IUPAC recommend that 'automation' be reserved for those systems involving feed-back loops. This is logically sound, but like many other definitions which have followed the growth of a subject it is likely to take some years before the distinction is adopted in common usage. The motivation for research and development in automation and/or mechanization was largely two-fold, to improve the cost-effectiveness in discharging large analytical workloads, especially those of a repetitive nature, and to improve method performance, notably precision, in these circumstances. The term 'automation' has been commonly used to describe the advances made.

The principal operations comprising a chemical analysis are as follows:—

1. Sampling

*Paper originally presented in the symposium *Automation Comes of Age* at the Pittsburgh Conference, Cleveland, 1978.

2. Sample pretreatment
3. Sample measurement, including standardization and calibration
4. Control of instrument parameters
5. Calculation of results, initially for the analytical result and then for the sample itself
6. Report generation, distribution and archiving

In considering the potential benefits of automating an analysis each item above should be considered, although in many instances sampling will be an external operation not amenable to automation. The majority of publications in the field of automatic analysis have dwelt on particular facets of the total subject. Recently attention has been focussed on the post-analysis stages, and data processing aspects, using computers, microprocessors or simple calculators, have been extensively studied. Unfortunately data processing has assumed too much importance in the context of automation and this imbalance must be redressed quickly.

In planning the introduction of automation into an analytical laboratory it is important not only to consider all stages of the analysis but the wider context within which the laboratory serves the organization of which it is part. In this way critical considerations can be highlighted and priorities identified. These could include the need to collate results of multiparameter analyses before reporting or, in clinical analysis, the preservation of sample-patient identification.

Research into automation of analytical techniques is prompted by the need to produce cost-effective solutions to an ever-increasing demand for chemical analysis. The analytical chemist plays an essential role in providing basic and control information in the chemical and manufacturing industries, and the increasing degree of legislative control serves to emphasise this function; for example in the United States it is necessary to label all foods with their vitamin content. There is growing concern for the quality of the environment, both in general and in the work place, and this has also increased the demands on the analytical chemist. Ultimately the public bears the cost of much of this analysis and it is therefore important to provide facilities for economic and efficient discharge of the requirements while maintaining the quality of the measurements made. Automation and mechanization are clearly consistent with these principles and in order to satisfy the demand adequate techniques for sample handling, measuring and reporting of results must be devised.

There is little published by way of performance data of routine automatic instruments, even in the clinical areas it has often been difficult to obtain proper inter-laboratory assessments of instrument performance, and the rate of development of 'new' instruments far exceeds the accumulation of meaningful performance characteristics on existing ones. To some extent this situation arises because there is no single international committee or organisation co-ordinating efforts in this area, which is perhaps not surprising in view of the breadth of the subject area.

The circumstances under which an instrument is operated are also extremely varied. In the extreme case it may be used by non-scientific staff to control a process plant in a hostile environment and the design criteria for instruments of this type are of necessity far more demanding than for those used in a clean laboratory by a graduate scientist. Further restrictions to progress in the use of automation are imposed by the fact that commercial companies currently largely define their own market areas, so that the development and marketing costs are spread. In the clinical area this has been less limiting since the analytes and sample matrices are not as varied as in the industrial sector.

### Developments in the philosophy of automation

As already stated, the overriding benefits of automation are economic in nature. The rapid introduction of automation into clinical laboratories and the large market offered to potential manufacturers have prompted many developments in instrumentation. Far less consideration has been given to the overall philosophical approaches to the subject. Currently one can consider these developments in three categories (a) the approach where methodologies are fitted to the available techniques as in continuous flow analysis, (b) a total systems approach developed at the Laboratory of the Government Chemist which attempts to define the precise analytical requirement in toto, including transmission of the result to the customer, and which uses available techniques to solve the problem in the most effective manner and (c) the approach described by Arndt and Werder [5] where a systems study is carried out on the available manual procedures which are then broken down into a number of steps, a complete rationalisation of these steps then generates a flexible range of equipment designs to meet the varied needs of an analytical laboratory. While the first approach typifies that followed by the majority of the instrument companies, the second is obviously the province of the systems designer working in a multidisciplinary laboratory. The third approach offers a fresh and encouraging input from one of the world's major instrument companies embarking on an automation programme in concert with an industrial chemical company already committed to the principles and benefits of automating their work pattern.

### Continuous, flow injection, discrete and centrifugal analysers

Since the availability of commercial instrumentation influences most of the work of research groups, the merits of the various techniques will briefly be reviewed. While the majority of samples in the liquid state are analysed automatically by one of two approaches, discrete or continuous, lately two other approaches have found favour, centrifugal and flow injection methods. In the discrete method each sample is maintained as a separate entity and placed in a separate receptacle, the stages of dilution, reagent addition and mixing are performed separately by mechanically transporting the sample to dispensing units where controlled additions are made individually to each sample. Each treated sample is then presented in turn to the measuring unit. Normally the discrete analytical system processes a batch of samples sequentially either by collecting a batch on a turntable or by mechanical transport direct to the next module. In continuous flow analysis the sample is converted into a flowing stream by a pump and the necessary reagent streams. Ultimately the treated sample is pumped to a flow-through measuring cell unit and thence to waste.

Centrifugal analysers treat a batch of samples in parallel and use centrifugal force to mix solutions and transfer them to cuvette rotors which intercept the light beam of the photometer as they rotate. The basic principles of the system, often called GeMSAEC (General Medical Sciences — Atomic Energy Commission) and developed at Oak Ridge National Laboratory USA, are described by Anderson [2]. Sample and reagent holders are fabricated in Teflon as is the cuvette rotor in which the photometric measurement is made. The holders are designed in such a way that no mixing of sample and reagent occurs when the apparatus is stationary but on rapid rotation good mixing occurs, the speed of the rotor is then reduced, while the measurements are made. The instrument, for which a dedicated computer is a necessity, can be used for kinetic as well as static measurements. The main disadvantage of the centrifugal approach is that it requires an additional dispensing unit to fill the individual rotors. This is not simple to provide but it is essential if the inherent advantage of speed of analysis is to be ensured.

Flow injection analysis [3] has lately received renewed attention. It is a very simple modification of continuous flow analysis which utilises rapid injection of an aqueous sample, either through a syringe, septum or a sample loop, into a continuously moving stream of a reagent. The simplicity is illustrated by Figure 1, a schematic diagram for an analysis using an ion selective electrode. The injected sample forms a zone which is transported towards a sensor which continuously records the change in analyte concentrations, using, for example, absorbance or electrode potential. As the sampling

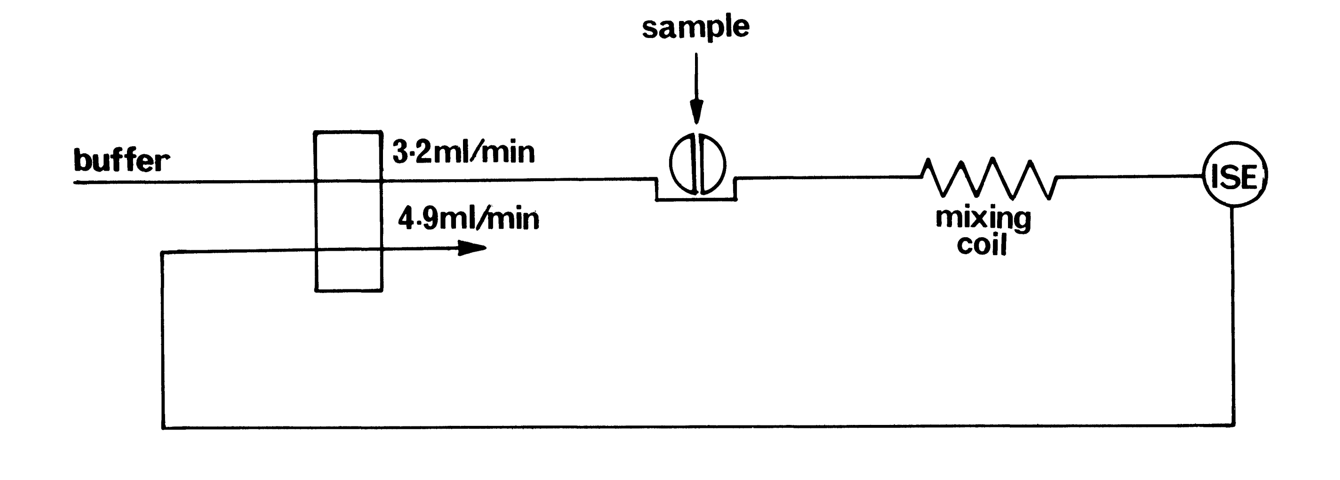

*Figure 1. Schematic arrangement for a simple flow injection analysis using an ion selective electrode.*

time is short the sampling rate can be high, but the major advantage is in speed of response to an analytical change which therefore makes the flow injection principle suitable for process control. In contrast to conventional AutoAnalyser methodology air segmenting is not required. The approach has been exploited by Ruzicka and co-workers, [3, 6] and its advantages and limitations are described by Stewart et al [7]. Simple reactions may be transferred directly from a manual into a flow injection procedure and the incorporation of separation procedures such as solvent extraction has recently been reported [8].

Discrete methods retain the sample as an entity and cross contamination is effectively eliminated, the fate of a sample at any time is known and it is difficult to confuse one sample with another. In a continuous system many samples are often being processed at any one time and although there is usually no difficulty in assigning sample identity, indeed some systems include a means of sample identification, problems can arise where successive samples have similar or zero responses. Insertion of frequent standards affords regular datum points. Unless precautions are taken, interaction can occur between successive samples; in general this is minimised or avoided by altering the time sequences and by reducing the sample rate. Where this reduction in speed is unacceptable some attempts can be made to compensate for sample interaction using computer data processing techniques but this is generally not desirable.

Usually continuous systems are simpler to design, operate and maintain. Sample transport and reagent additions are carried out using a single peristaltic pump. Many analytical methods can be incorporated into AutoAnalyser technology using essentially similar modules. The Multiple Test cartridge recently introduced by Technicon allows a series of tests to be designed on to a single analytical cartridge and rapid changeover from one test to another is facilitated using a multiple switching valve. For a laboratory having a limited sample input but a requirement for a range of analytes the system may well represent an economic purchase. Discrete analysers are complex and each reagent or sample requires its own dispenser. Chemical or physical separation techniques such as precipitation, distillation and solvent extraction are not readily available with commercial discrete systems and where these are needed continuous analysers are favoured. Continuous methods are limited by the use of flexible pump tubes; certain corrosive and reactive materials cannot be accurately pumped, although advances are being made in the development of inert plastics. This restriction does not apply to discrete methods where there are fewer constraints on the material used for construction.

The chemistry of the method and the required rate of analysis will largely determine which instrumental approach is adopted although continuous and discrete philosophies are not mutually exclusive. This is one reason why a multidisciplinary approach to automation commends itself as an effective means of answering the first and most vital question, how should a new problem of automatic analysis be approached? Too often the advocates of one approach have failed to see the virtue of the others, however each should be evaluated in the context of the problem. Recently, Technicon have introduced a discrete analytical system, the STAC system (Single Test Analyser with Computer), this closes the loop with regard to the continuous flow analyser. This bodes well for the future that the philosophy adopted will be matched to the analytical requirement.

### Total system approach to laboratory automation

The total systems approach is the one which has evolved over the last few years at the Laboratory of the Government Chemist. A multidisciplinary team is required to design and develop automatic systems but it must be emphasised that an appreciation and understanding of the analytical chemistry involved is of paramount importance. Figure 2 shows the structure of the automation team at the Laboratory of the Government Chemist and attempts to highlight the individual group functions. It is not, of course, obligatory that all such functions are undertaken inhouse, but automation is a systems problem. Almost all the projects that are considered for automation rely heavily on a close cooperation of specialists in the various groups as well as the analysts involved. A successful automatic analyser is often achieved through a blend of analysis, electronic control and data processing and this necessitates a working expertise in each discipline.

The reasons for setting up a specific group internally are shown schematically in Table 1. However, this is further illustrated by a direct example of the fact that the Laboratory's

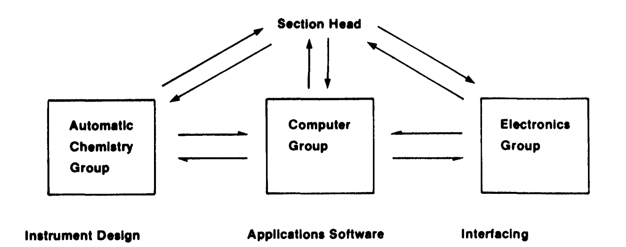

*Figure 2. Schematic representation of Automation Group structure at the Laboratory of the Government Chemist.*

**Table 1 Reasons for setting up an in-house automation team at the Laboratory of the Government chemist**

| | |
|---|---|
| 1 Economics | — Improve the speed of analysis to undertake survey work.<br>— Free staff from routine chores to do real science.<br>— to obtain reproducible results in long runs of samples. |
| 2 The requirements for automation are complex | — Need to automate is compelling but solutions not obvious.<br>— Requirements not met by instrument manufacturers for example in gas chromatography. |
| 3 Specification | — Most difficult operation in an automation project, team acts as an interface between users and designers. |
| 4 Operation, maintenance and upgrading of instrumentation | — Operation in itself does not require highly skilled and trained staff but other aspects do, a team inhouse can most quickly react to changing situation. |

requirements have not been in the past met by the instrument companies themselves. Instrument companies have been in the main concentrating on measurement techniques. In this laboratory samples generally require considerable pretreatment and chemical treatment prior to measurement and samples are not generally suitable for immediate injection on to a gas chromatograph.

By 1970 the problems relating to automatic gas chromatograph had been identified and a system devised by Stockwell and Sawyer [9], which included a sample preparation system based on continuous flow principles, was interfaced to an analytical gas chromatograph. The instrument described specifically analysed a range of tinctures and essences for their ethanol content but the applicability of this type of system is restricted only by the ability of continuous flow principles to cope with the materials being analysed. It was only recently that the demands of sample preparation and the time taken were recognised and a complete analytical system designed for the analysis of vitamin tablets using similar principles to that described for gas chromatography was designed and developed by Burns [10].

The total systems approach attempts to view the complete analytical problem from sampling through to reporting, and indeed further if desirable, and it is not limited by the individual analyst's needs or those of the laboratory. Such an approach is illustrated by reference in this paper to work in the author's laboratory. It is important that the analyst is not compromised in any way or limited in performing his tasks by any inadequacies of the automatic system. The solution may not be an all encompassing instrument but if a careful economic and scientific assessment is carried out the level of automation provided should match the problem and maximise the benefits from its implementation. If a simple dispensing system or mechanical cycling system is the most logical solution then this is provided and the temptation to develop more extensive automation is resisted. The development of a temperature cycling

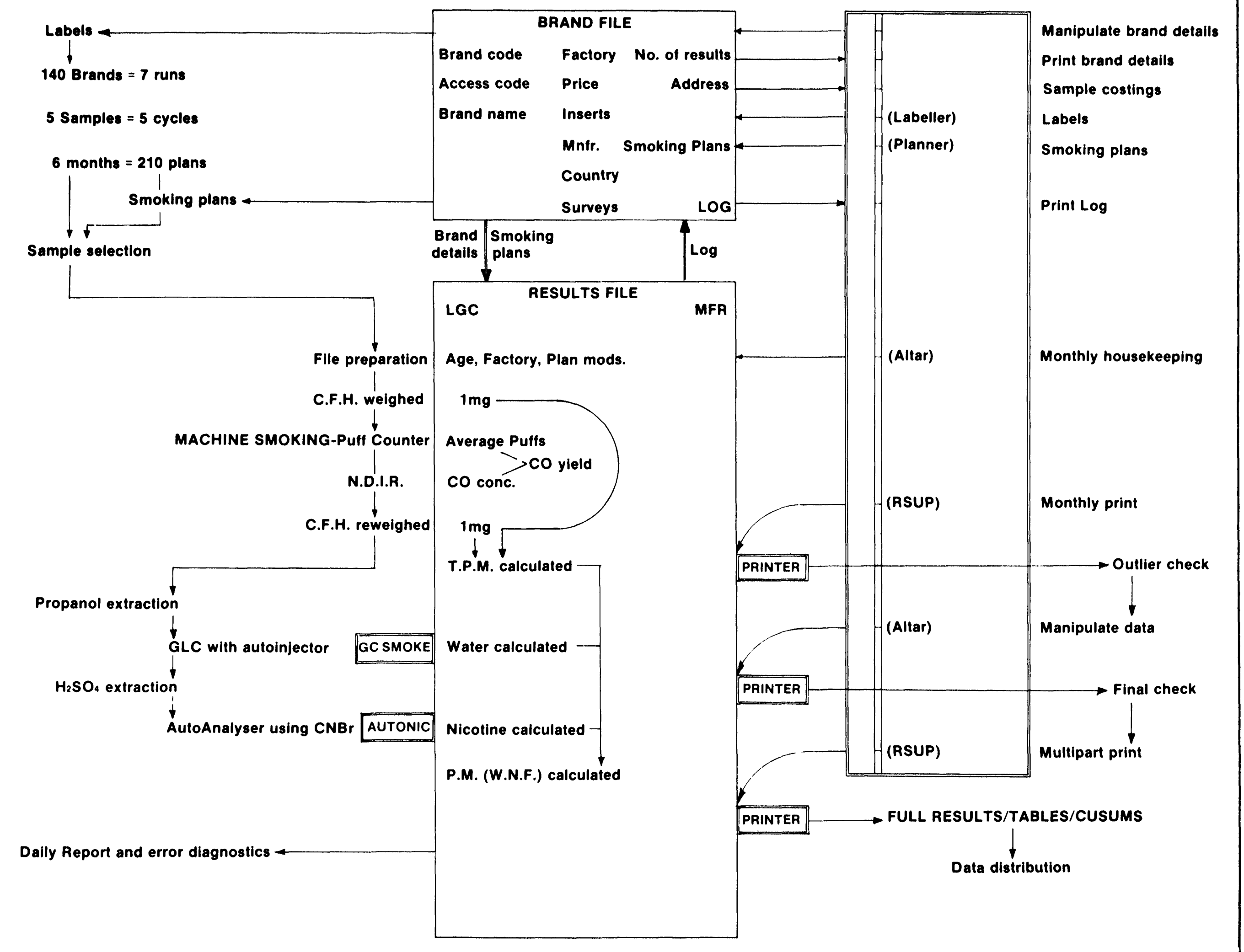

*Figure 3. Diagramatic representation of the relationship between the automatic analytical procedures, the data files and the large interactive control program BRANDER, C.F.H.=Cambridge Filter Holder; T.P.M.=Total Particulate matter; N.D.I.R.=Non dispersive infra red; P.M.(WNF)=Particulate matter water and nicotine free; ▢=Computer program function.*

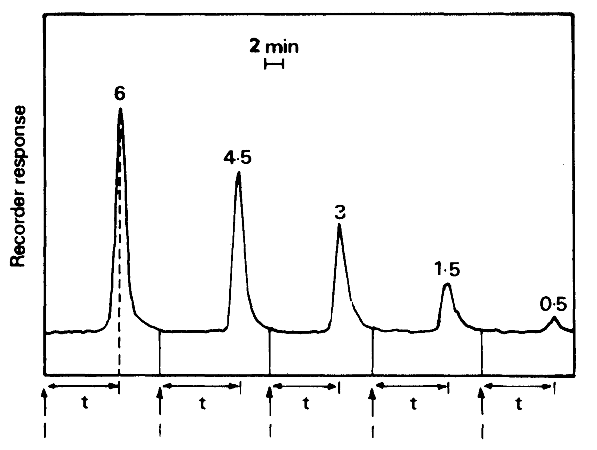

*Figure 4. A typical recorder showing a range of standard injections in the range 0.5 to 6.0 mg kg$^{-1}$ of furfural. I, injection point; and t, retention time*

instrument to test the effectiveness of various dental materials serves to illustrate this point [11]. In other situations, such as the tar and nicotine analyses of cigarettes available in the UK, the complete function of sampling, analyses and reporting have been automated. The object of the survey carried out on behalf of the Department of Health and Social Security is to provide a league table ranking the brands of cigarettes available in the UK market in a tar and nicotine context order. Cigarettes are smoked on a 20-channel smoking machine, the tar produced for each brand is measured, the water and nicotine (as total nicotine alkaloids) contents of the tar are then determined, and these values subtracted from the tar value to provide a dry tar figure which is then the basic ranking parameter used. The analytical procedures involve automatic moisture determinations, in addition the automatic recording of the puff count (a quality controlling parameter) and the automatic recording of the carbon monoxide level of each of the cigarettes are integrated into an overall plan for automation. How these analytical procedures are linked into the data handling and reporting software is illustrated in Figure 3. Several stages of reporting are required and the computer is involved throughout the analytical procedure. In the initial stages it is important to randomise the cigarettes between the day of analysis and the individual channel to which they are smoked so that the computer prepares a smoking programme which prompts the analyst to load up the machine in the correct order and this order is in fact used throughout the analytical processes to locate any particular sample within any particular run. In this manner it is possible to avoid any complicated sample identification procedures.

The analytical processes have not been restricted by any one technique for in this particular application continuous flow principles have been utilised for the determination of total alkaloids, the discrete approach for the analysis of water by gas chromatography and online analysis principles for the determination of carbon monoxide in cigarette smoke. In this requirement the automation group has been involved from the outset. This overcomes many of the problems experienced when attempting to automate analytical methods which have been used in a manual regime for a number of years. In these cases it is often difficult to discern the true analytical requirement from the accepted manual approach. The basic experience of an automation group in the skills of analytical chemistry and its understanding of the aims and objectives of the laboratory itself are invaluable. This understanding would be lost by contracting the automation project outside the organisation either to a commercial instrument company or a consulting laboratory. In some situations this close understanding and the ability to involve the users to define the precise analytical needs has avoided the mechanization of a single manual procedure which involved complicated solvent extraction procedures. These are difficult to automate. Instead the concept of hybrid chromatographic analysis has been evolved. Stockwell and Lidzey [12] have described the coupling of a simple gas chromatography separation to a specific colorimetric reaction specifically to identify and measure furfuraldehyde in a gas oil sample. The resultant chromatogram for samples having furfuraldehyde present shows a single peak at a specific retention time in marked contrast to a conventional flame ionisation detector response of a similar sample. Figure 4 shows the simple chromatogram produced for a range of standard samples of marked oils, a single peak is obtained for each sample containing furfuraldehyde.

### Automated individual analysis in wet chemistry

In contrast to the total systems approach, the approach described by Arndt and Werder [5] involves close co-operation of a commercial instrument company with a laboratory attached to a large industrial chemical organisation. The analytical work load in industrial wet chemistry laboratories is characterised by a multitude of procedures and methods as well as small numbers of samples in serial runs of analyses. For this reason a large sector has resisted automation and a new approach is needed. This has emerged as a modular sub-division of manual analytical procedures into basic operations. These are assigned executive parameters that determine the detailed operation. From this it is possible to derive a conceptual design of an automatic analysis system with the following characteristics: it is possible to run individual samples and small and large series of samples, each sample is contained in its own cup, the automated units are self-cleaning and existing analytical procedures may be used. A complete range of instruments is available based on these principles from Mettler AG, individual instruments tailored to individual laboratory's own requirements are easily configured. These are based either on control from a desk calculator or for more sophisticated requirements use a hierarchical-system of computers to control individual modules and to coordinate control and reporting.

This approach represents a significant advance on the philosophy adopted by instrument manufacturers; once the credibility of the approach has been proved in industrial laboratories it will be interesting to see how far it will merge with the total systems philosophy and attempt to solve problems directly rather than mimic manual procedures.

### Conclusions

Over the last few years a considerable number of studies have been made in developing new automatic instruments and these, coupled to the effective implementation of computer power, particularly microprocessors, predict a healthy future. Seemingly, almost any analytical problem can be solved and automation achieved. Some of the more philosophical constraints such as management, training and education have already been touched on in this paper — what then are the remaining influences that will constrain the future of automation? The most difficult and time consuming aspect is very often the sample preparation stage and ways of avoiding this are obvious goals for the future. However, more efforts in mechanising the existing procedures, i.e. new pumps, dispensers, solvent extraction systems must be developed. With regard to the former goal, the development by Norris et al [13] of the Infra-Alzyer technique to analyse samples of grain for oil, moisture and protein, whilst slow to develop commercially, has many potential applications, such as tobacco analysis. Malmstadt and co-workers [14] are actively working in this latter area, and the introduction of the vitamin assay system by Burns [10] shows that commercial interest is also alive in this aspect. Two other facets must, however, be considered in this respect. On the one hand a limitation of the automation approach may be caused by insufficient knowledge of the chemistry involved in any process. Roy and co-workers [15]

have considered this aspect in some detail for the analysis of vitamins. Accepted manual methods often do not correlate well with automatic analysis primarily because the operating conditions employed in a manual analysis lack the control of the automatic systems. The other significant problem is that components used in commercial instruments or which are readily available are not sufficiently inert to handle corrosive materials often required for an automated procedure. In the automated digestion system described by Jackson et al [16], where concentrated nitric and sulphuric acid are used to destroy the organic matrix of foodstuffs prior to trace metal analysis several components had to be re-designed and developed in-house [17-19]. In addition, a unique displacement pumping arrangement was devised to introduce a slurry of partially digested food quantitatively into the spinning digestor helix. As with the work of Roy [15] with vitamins an in depth appreciation of the reactions involved was also essential to complete the programme of work and to obtain meaningful results.

This paper has centred on the problems of automation in the area of industrial analytical chemistry, however, it is clear that whilst being a multidisciplinary subject, automatic chemistry also transcends disciplinary boundaries. There seems considerable merit therefore in the cross fertilisation of ideas and concepts between the clinical chemists, the process control chemist and the industrial chemist. Such transfer of ideas and evolvement of an acceptable philosophy can only be of benefit to all concerned, and will assist the development of unified philosophies of automatic analysis.

REFERENCES

[1] Skeggs, L. T., *American Journal of Clinical Pathology*, 1957, **28**, 311.

[2] Anderson, M. G., *American Journal of Clinical Pathology*, 1970, **53**, 778.

[3] Ruzicka, J. and Hansen, E. H., *Analytica Chimica Acta*, 1957, **78**, 31.

[4] Deans, D. R., *Proceedings of The Analytical Division of The Chemical Society*, 1977, **14**, 199.

[5] Arndt, R. W., Werder, R. and Fres, Z., *Analytical Chemistry*, 1977, **278**, 15.

[6] Ruzicka, J., Hansen, E. H. and Reetz, B., *Analytica Chimica Acta*, 1977, **89**, 24.

[7] Stewart, K. K., Beecher, G. R. and Hare, P. E., *Analytical Biochemist*, 1976, **79**, 162.

[8] Karlberg, B. and Thelander, S., *Analytica Chimica Acta*, 1978, **89**, 1.

[9] Stockwell, P. B. and Sawyer, R., *Analytical Chemistry*, 1970, **42**, 1136.

[10] Burns, D. A., Seventh Technicon International Congress, New York 1976, *Proceedings* 1977.

[11] Morley, F. and Stockwell, P. B., *Journal of Dentistry*, 1977, **5**, 39.

[12] Lidzey, R. G. and Stockwell, P. B., *The Analyst*, 1974, **99**, 749.

[13] Norris, K. H., and Hart, J. R., *Proceedings 1963 International Symposium on Humidity and Moisture*, Peinhold, New York, 1965, **4**, 19.

[14] Renoe, B. W., O'Keefe, K. R. and Malmstadt, H. V., *Analytical Chemistry*, 1976, **48**, 661.

[15] Roy, R. B., Conetta, A. and Salpeter, J., *Journal of The Association of Official Analytical Chemists*, 1976, **59**, 1244.

[16] Jackson, C. J., Porter, D. G., Dennis, A. L. and Stockwell, P. B., *The Analyst*, 1978, **103**, 317.

[17] Porter, D. G., Jackson, C. J. and Bunting, W., *Laboratory Practice*, 1974, **23**, 111.

[18] Lidzey, R. G., Jackson, C. J. and Porter, D. J., *Laboratory Practice*, 1977, **26**, 400.

[19] Jackson, C. J., Morley, F. and Porter, D. G., *Laboratory Practice*, 1975, **24**, 23.

### High-speed, automatic dispenser/diluter or dual pipetter/mixer

**David L. Krottinger***
**Michael S. McCracken****
**Howard V. Malmstadt†**

*School of Chemical Sciences, University of Illinois at Urbana-Champaign, Urbana, Illinois 61801, USA.*

**ON** several of our automated analytical systems [1, 2, 3] we have need for a device that can either operate as a precise dispenser/diluter of discrete quantities of solution or can be used to pipet automatically both a sample solution and a reagent and efficiently mix them as the sample-reagent solution is rapidly and efficiently mix them as the sample-reagent solution is rapidly delivered into a measurement cell. Although there are commercial systems on the market, none have the combination of high-speed refill/delivery/mixing, modest cost, and automated features that we require. Consequently, we have developed a portable stand-alone unit that refills, delivers and mixes solutions in ways quite similar to some stopped-flow instruments. Experience with this type of automated unit for more than one year has provided ample data to demonstrate its general utility and reliability for the analytical laboratory. Therefore, we present here the details of the unit, which constructed primarily from commercially-available parts, and also some test results to indicate its performance characteristics.

*Present Address: Continental Oil Co., Analytical Research Section, Ponca City, OK 74601.

**Present Address: The Proctor & Gamble Co., Miami Valley Laboratory, Cincinnati, OH 45200.

†Address correspondence to this author.

### Instrumentation

The design of the dual pipetter/mixer or dispenser/diluter is based on the principles illustrated in Figure 1. Pneumatically-operated syringes together with pneumatically-actuated, 3-way KEL-F valves enable the syringes to be filled and the solutions to be delivered by electronic control signals, and an X-Y mixer in the delivery channel to the measurement cell provides the mixing of the two solutions.

The completely assembled unit is shown in Figure 2, and the partially-assembled unit showing the framework for mounting the syringes and valves and drive system is shown in Figure 3. An exploded view of the unit illustrating the interconnections of each of the components is shown in Figure 4 with the components that were used in the construction listed in Table 1. More detailed description follows.

### Dual, 3-way valves

Two commercially available, 3-way valves were used in the unit to provide for automatic control of the solution flow. Both 3-way valves contain an inlet connection to the solution reservoir, a syringe port in which the desired syringe assembly is mounted, and an outlet port for the solution to be delivered from the syringes. A KEL-F slider is pneumatically positioned in each valve to interconnect the desired ports. These valves have an

